# Supplementary material for: Hypoxic pulmonary vasoconstriction as a regulator of alveolar-capillary oxygen flux: A computational model of ventilation-perfusion matching
Source: PLoS Comput Biol. 2021 May 6;17(5):e1008861. doi: 10.1371/journal.pcbi.1008861 (PMC8130924; doi:10.1371/journal.pcbi.1008861)
Supplement: S1 Text — (PDF) [file pcbi.1008861.s001.pdf]

Network Morphometry S1 Table summaries the the morphometry of the generated vascular networks. The information in the bottom block of this table corresponds to the data plotted in Fig 3.

Airway model In contrast to the detail of our vascular networks, we use a relatively simplified airway model. S1 Fig illustrates how the airway and alveolar compartments are connected to each other.

Effect of tidal volume on oxygenation To understand how tidal ventilation affects gas exchange in our model, we adjust the parameter Q_V^* which represents bulk airflow. Tidal volume (TV) is proportional to airflow ($TV/Q_V^* = 0.272$), so increasing this parameter increases tidal volume, and decreasing this parameter decreases tidal volume. S2 Fig shows how tidal volume impacts oxygen uptake. Increasing tidal volume increases oxygen uptake in a manner that asymptotically approaches oxygen partial pressure in the air (150 mmHg).

Effect of HPV and uniform vasoconstriction on regional oxygen tension distribution. While vasomotion does alter the distribution of blood flow, another functional consequence of this action is to alter the distribution of oxygen in the alveolar and vascular space. S3 Fig shows the distribution of oxygen tension in network A under conditions of no regulation, with regulation from HPV, and with uniform vasoconstriction.

Effect of airway occlusions on the distribution of vascular tone from HPV. In Fig 10, we show airway occlusions affect the distribution of hemodynamics, V/Q matching, and oxygen flux. In S4 Fig, we show the underlying changes in tone from HPV that mediate the changes in blood flow, V/Q matching, and oxygenation
