# Supplementary material for: Hypoxic pulmonary vasoconstriction as a regulator of alveolar-capillary oxygen flux: A computational model of ventilation-perfusion matching
Source: PLoS Comput Biol. 2021 May 6;17(5):e1008861. doi: 10.1371/journal.pcbi.1008861 (PMC8130924; doi:10.1371/journal.pcbi.1008861)
Supplement: S1 Table — Descriptive statistics for each vascular network. Quantities are presented as mean ± standard deviation. The top block summaries the size of the perfusion zones, and the bottom block summarizes the diameters and lengths for each diameter-defined Strahler order. (PDF) [file pcbi.1008861.s002.pdf]

Table 1. Pulmonary vascular network and perfusion zone morphometry Descriptive statistics for each vascular network. Quantities are presented as mean ± standard deviation. The top block summaries the size of the perfusion zones, and the bottom block summarizes the diameters and lengths for each diameter-defined Strahler order.

Network	A			B			C		
Area of perfusion zone (μm^2)	907.34 ± 227.68			594.19 ± 150.69			639.47 ± 165.07		
dd Strahler Order	Segments	Diameter (μm)	Length (μm)	Segments	Diameter (μm)	Length (μm)	Segments	Diameter (μm)	Length (μm)
12	1	540.53	2,250.00	2	600.90 ± 0.67	525.00 ± 300.00	3	589.10 ± 0.83	625.00 ± 304.14
11	4	533.91 ± 2.28	375.00 ± 75.00	26	485.95 ± 3.63	452.88 ± 51.88	8	568.70 ± 1.92	487.50 ± 40.09
10	5	510.21 ± 4.13	405.00 ± 137.48	29	360.15 ± 4.44	375.00 ± 39.74	4	517.90 ± 1.35	450.00 ± 61.24
9	14	385.69 ± 6.42	514.28 ± 72.85	32	255.61 ± 3.36	473.44 ± 40.75	21	407.19 ± 4.47	442.85 ± 52.25
8	34	266.08 ± 4.20	555.88 ± 47.62	136	185.18 ± 1.14	266.91 ± 20.35	29	322.25 ± 3.61	411.21 ± 40.37
7	89	185.16 ± 1.41	317.70 ± 30.53				22	253.83 ± 4.09	507.95 ± 52.21
6							122	184.13 ± 1.11	288.32 ± 18.47
