# Supplementary figures and images for: Hypoxic pulmonary vasoconstriction as a regulator of alveolar-capillary oxygen flux: A computational model of ventilation-perfusion matching
Source: PLoS Comput Biol. 2021 May 6;17(5):e1008861. doi: 10.1371/journal.pcbi.1008861 (PMC8130924; doi:10.1371/journal.pcbi.1008861)

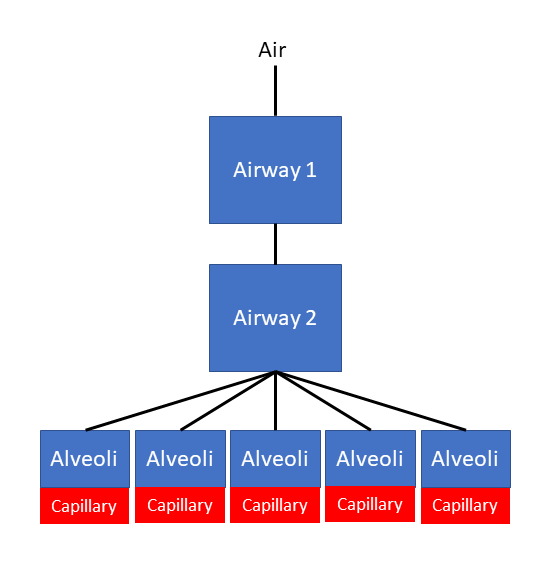

Supplement: S1 Fig — Blue boxes denote compartment in our airway model. Airway 1 interacts with the atmospheric air and airway 2. Airway 2 functions as a mixing chamber and interacts with all of the alveolar compartments. Each alveolar compartment exchanges oxygen with a capillary compartment. (TIF) [file pcbi.1008861.s003.tif]

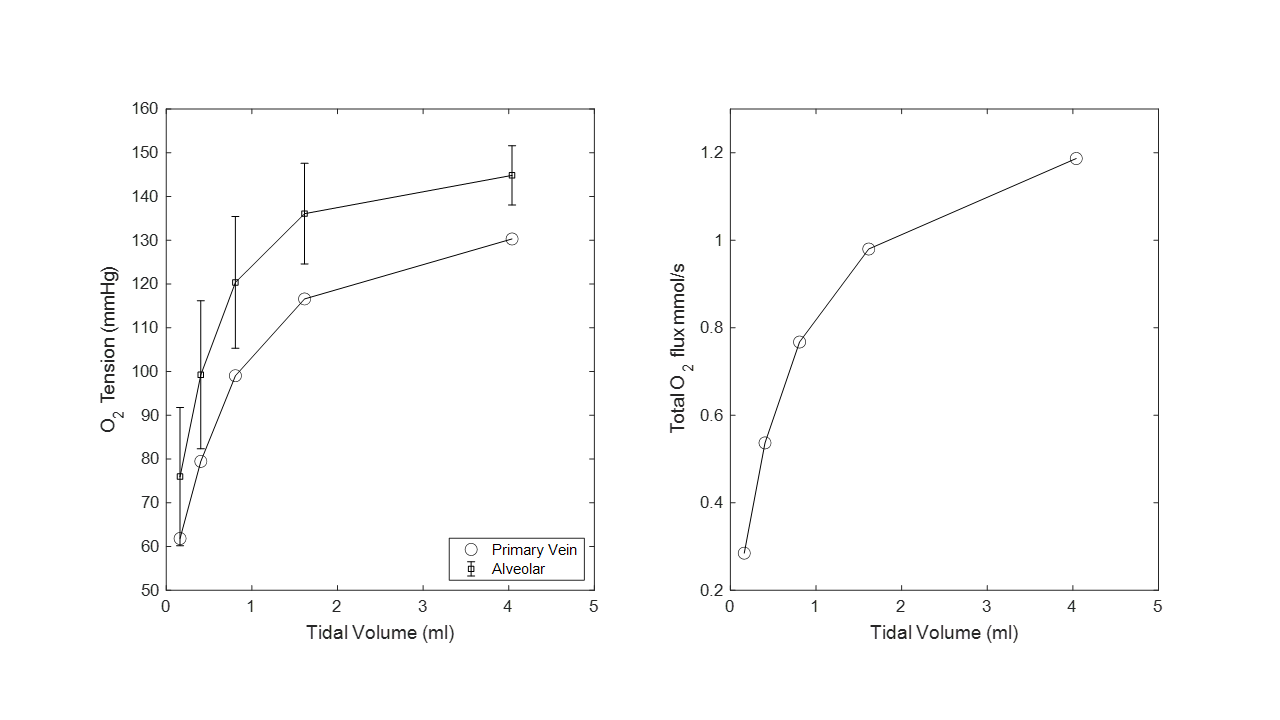

Supplement: S2 Fig — (A) Oxygen tension in the primary vein and mean and standard deviation in the alveolar compartments as a function of tidal volume; (B) Total oxygen flux as a function of tidal volume. (TIF) [file pcbi.1008861.s004.tif]

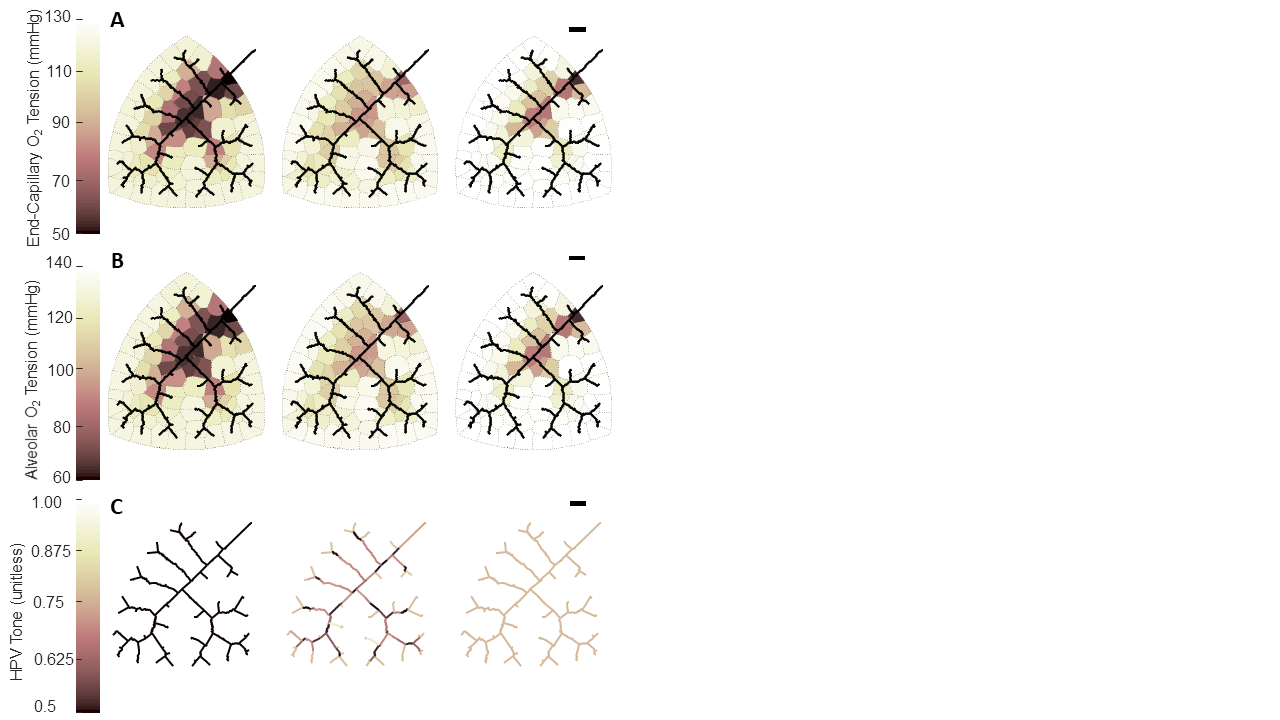

Supplement: S3 Fig — Leftmost column of networks are without regulation from HPV, middle column of networks are with regulation from HPV, and the rightmost column of networks are with uniform vasoconstriction, where T* = 0.8 is used for the global vascular tone. The black scale bar is 1000 μm. (A) End-capillary oxygen tension; (B) Alveolar Oxygen tension; (C) Tone from HPV. (TIF) [file pcbi.1008861.s005.tif]

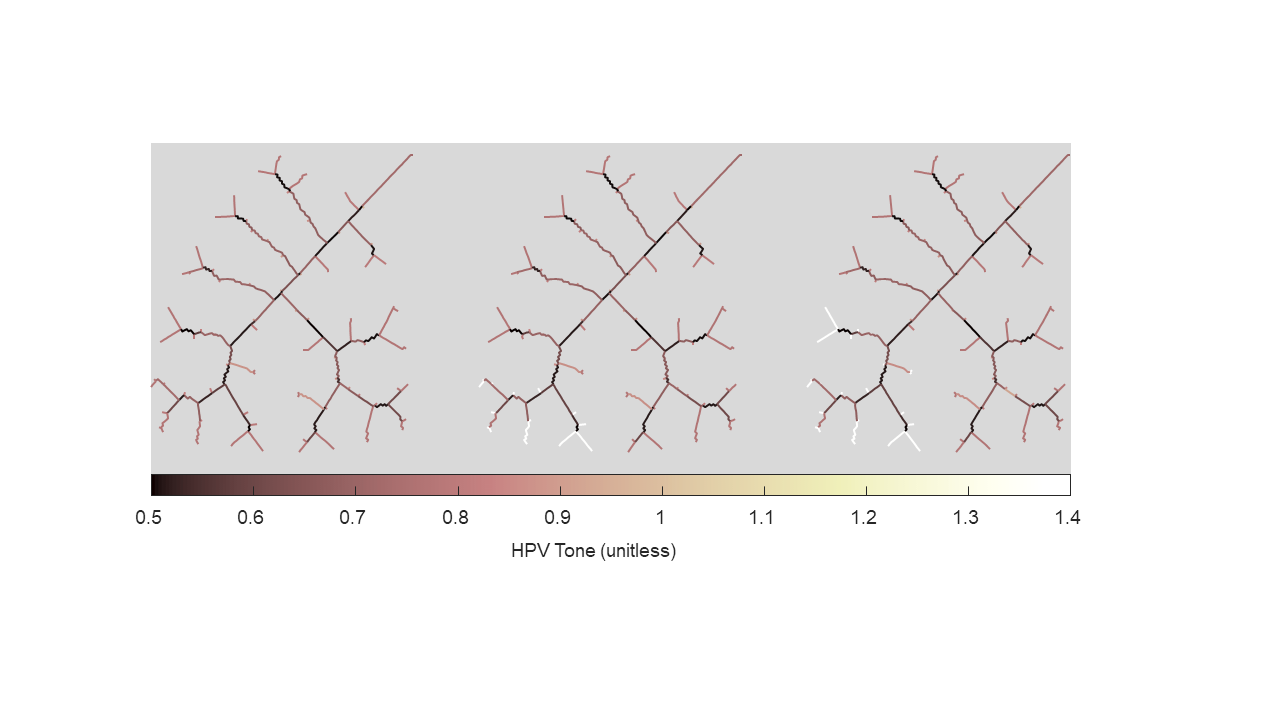

Supplement: S4 Fig — This figure corresponds to simulations shown in Fig 10. Leftmost network is with 0% of the alveoli occluded, middle network is with 17% of the alveoli occluded, and the rightmost network is with 28% of the alveoli occluded. The first airway occlusion is at the most southwestern perfusion zone, and progressive airway occlusions are made following the structure of the vascular network. (TIF) [file pcbi.1008861.s006.tif]
