# Supplementary material for: Hypoxic pulmonary vasoconstriction as a regulator of alveolar-capillary oxygen flux: A computational model of ventilation-perfusion matching
Source: PLoS Comput Biol. 2021 May 6;17(5):e1008861. doi: 10.1371/journal.pcbi.1008861 (PMC8130924; doi:10.1371/journal.pcbi.1008861)
Supplement: Attachment — Submitted filename: responses_to_reviewer_comments.docx [file pcbi.1008861.s007.docx]

**Answer to Reviewer #1**

The authors describe the development of a gas exchange model that accounts for HPV and makes predictions regarding blood flow and gas exchange at various scales. The geometric and gas exchange parameters of the 2D model are selected/fitted to correspond to nominal conditions in the rat lung. The microcirculation is not explicitly represented. The model predicts homogenization of blood flow and oxygen transport at smaller scales, leading to overall improvement in oxygen uptake at the level of the whole lung. The authors are to be commended for providing access to their code.

We thank the reviewer for their time and acknowledging our efforts to make our model accessible and reproducible.

GENERAL COMMENTS

Please consider adding scale bars to figures including lung lobes - how large was a typical gas exchange unit?

Thank you for this suggestion - we have added scale bars to Figures 6, 7, and 8.

Spatially, gas exchange units occupy 907.34 +/- 227.68 um^2 for Network A, 594.19 +/- 150.69 um^2 for Network B, and 639.47 +/- 165.07 um^2 for Network C.

We have summarized this information and descriptive statistics regarding the morphometry of the vascular networks in **Table A1** of the revised manuscript.

To what extent does tidal ventilation affect gas exchange?

We can adjust tidal volume by adjusting the value of the parameter Q*_V, which controls the magnitude of bulk airflow. We plot alveolar and end venous oxygen partial pressure, and total oxygen flux as a function of tidal volume. Increasing tidal volume increases oxygen uptake in a manner that asymptotically approaches oxygen partial pressure in the air (150 mmHg).

Lines 724-729, Figure A2

What values were used for the conducted response length constant l_k?

If an arterial vessel segment has k-many perfusion zones (capillary segmetns) downstream, then l_k is the path length from the k-th perfusion zone to the midline of the vessel segment. These are static numbers computed from the structure of the vascular networks. If there are j-many segments in a network, then there are j-many unique sets of l_k values.

The parameter lambda governs the spatial decay of the conducted response signal and is reported in Table 1.

To improve clarity, we have added the following sentences to the manuscript:

Lines 238-245

What is the range of vessel sizes simulated and the range of hematocrit values in the smallest vessels?

The average diameter and length of vessels simulated is illustrated in Figure 3D and 3E, respectively. To improve clarity, we have reported this information in **Table A1** in the revised manuscript:

We assumed a constant value for hematocrit for every vessel (0.4). We recognize that this is a strong assumption, and are aware of the phenomenon of phase-separation and uneven partitioning of the discharge hematocrit at bifurcations (Pries, A. R., Secomb, T. W., Gaehtgens, P., & Gross, J. F. (1990). Blood flow in microvascular networks. Experiments and simulation. Circulation research, 67(4), 826-834.). We did consider accounting for this level of detail during model development but choose to not as a strategy to reduce the complexity and computational expense of our model.

To provide greater clarity to this nuance, we have added the following sentences to the Discussion of our revised manuscript:

Lines 527-534

The authors should comment on the relative increase in total vascular resistance with HPV vs UVC, and to what extent this is a consequence of parameter selection (T values)?

We thank the reviewer for this suggestion. We have added a row to **Table 5** reporting the total vascular resistance for w/o HPV, HPV, and UVC. Additionally, we have added sentences to the Discussion about the similarities and differences between the effects of HPV and UVC on vascular resistance.

Lines 601-605

What technique was used to estimate the values of T selected for Figure 4?

The values of T^* used in Figure 4 was manually calibrated. We have clarified this in the text of the revised manuscript:

Line 322

Does the level of hypoxia selected correspond to the maximum sensitivity of HPV in the observed capillary/alveolar PO2 values?

The level of hypoxia selected corresponds to that which is experimentally achievable without immediately killing an animal or human subject. In the future, we plan to perform our own rat experiments to further scrutinize our model’s predictive capability. The HPV response is generally regarded to be dependent upon the degree of hypoxia, so presumably 0% oxygen (anoxia) would be the maximally sensitive point.

How do the observed values of CV compare to estimates in actual rat lung?

The CV of blood flow is dependent upon the sampling size of the region(s) observed. This is due to the fractal properties of blood vessel branching and blood flow in the pulmonary circulation (Glenny, R. W., Bernard, S. L., & Robertson, H. T. (2000). Pulmonary blood flow remains fractal down to the level of gas exchange. Journal of Applied Physiology, 89(2), 742-748.). In Glenny 2000, they report a blood flow CV of 0.35 when sampling 0.53 mm^3 volumes of rat lung and a fractal dimension of 1.12. The blood flow CV we report without regulation are approximately 2.2, and with regulation from HPV the CV becomes approximately 1.2. On average, our perfusion zones are 750 um^2 (0.75e-4 mm^2). The linear dimension of an average perfusion from our model is sqrt(0.75e-4 mm^2) = 0.027 mm, and we can extrapolate the volume to be (0.027mm)^3 = 2.05e-5 mm^3. Using the fractal relationship reported in Glenny 2000, we can extrapolate that a sampling volume of 2.05e-5 mm^3 corresponds to a CV of 1.01. Our predicted CVs are in the realm of reality but do overestimate the heterogeneity. This is likely due to the relatively small number of simulated perfusion zones – there are approximately 100 perfusion zones in each network, whereas the Glenny 2000 study uses 1000’s of sampling volumes when analyzing blood flow distribution in the entire rat lung.

We have updated the Discussion section to report this:

Lines 512-526

SPECIFIC COMMENTS

L153: Rationale for assumed value of hydraulic conductivity k

To our knowledge, hydraulic conductivity per unit length is not a quantity routinely measured or reported in the literature. Thus we treated k as an adjustable parameter and adjusted it to find a high quality fit to the data in Jiang et al (Jiang Z, Kassab G, Fung Y. Diameter-defined Strahler system and connectivitymatrix of the pulmonary arterial tree. Journal of Applied Physiology.1994;76(2):882–892.)

We have added these sentences to improve clarity:

Lines 140-141; 268-272; 490-497

Eq 19: Plus sign in denominator

We corrected this typo

L302: Hypoxic air fraction reads 21% rather than 10%

We corrected this typo

L336: Should read "proportional to its size"

We corrected this typo

**Answer to Reviewer #2**

The authors present a model for simulating the response of a network of blood vessels to hypoxic pulmonary vasoconstriction (HPV). The model includes a 2D network geometry, an oscillating ‘alveolar pressure’, a perfusion model, a gas exchange model, and a model for HPV. I have a number of concerns relating to presentation, assumptions, and lack of thorough analysis. The manuscript also needs a good going through for grammar and spelling, and accuracy in the equations and nomenclature. e.g. Palv is used variably for alveolar pressure and (as Palv,i) oxygen partial pressure in the alveoli.

We thank the reviewer for taking the time to thoroughly review our paper.

The introduction suggests that this will be a fully coupled model that will allow the authors to explore the impact of HPV on the integrated gas exchange system. But that does not come through in the results, and it is difficult to interpret from the methods as they are missing some important information (details below). The ‘integration’ between airways and vessels is minimal and is not fully described. A time-varying boundary condition for the blood flow model was not given, but I assume that the model was indeed time-varying? The only time-variation for the ‘airway’ model was an oscillation of an alveolar pressure. How does the dynamic blood pressure affect the model? How does the dynamic airway pressure affect the model? (the intro suggests that these are important).

We agree that the “integration” between airways and vessels is not fully described. This is the phenomena that we find interesting and wish to explore in deeper detail in the future, but currently exceeds the scope of this present study. We have removed this from our introduction to be clearer.

As the model is formulated as a system of ODEs, it is indeed time varying. For the analysis presented in the paper we use constant pressures at the arterial inlet and venous outlet to generate flow through the vascular network model. The model can be simulated using a pulsatile arterial pressure input, but in that case becomes too expensive to allow for parameter estimation. The use a constant inlet arterial pressure does not substantially impact the results.

Having blood flow be governed by ODEs enables regulation in the form of HPV to alter (regulate) the distribution of blood flow throughout the network. Given constant arterial inlet pressure, if the airway pressure is held constant, the model will equilibrate to a constant steady state. With a pulsatile airway pressure, the model achieves (pressure and flow) a period steady state.

The conclusion of homogenization of blood flow is not representative of the intact lung, where HPV increases heterogeneity of tissue perfusion. You have simulated regional PO2 for a uniform V, and allowed a non-uniform Q to adapt to this. The outcome is that Q becomes adapted to V (i.e. more uniform). However, in the intact lung the V distribution is highly non-uniform, so the conclusion that HPV ‘homogenizes’ Q is only relevant to the artificial scenario considered in the current model. Please interpret your results in the context of intact lung physiology.

This is an excellent point. The common wisdom is that HPV operates to regulate the distribution of blood flow so that it mirrors the distribution of air flow. To illustrate this, we performed additional simulations where we use occlude portions of the airway. We have updated the Results and Discussion section to report this:

Lines 440-461; 606-623; 701-710; Figure 10

The conclusion of increased transit time appears to be because the model predicts a reduction in cardiac output in response to increased resistance. I’m not convinced that this is realistic. And strangely this results in an increased PO2.

Thank you for pointing this out. This was something we were also concerned about and what motivated us to perform the uniform vasoconstriction (UVC) simulations as a control. Looking deeper into this, we realized that we did not normalize our RBC transit times properly due to bug in our code. We have corrected this **(Figures 6E, 7E, 8E)**

Looking at these correctly normalized RBC transit time distributions, we can appreciate that the normalized UVC transit time distributions are effectively superimposed upon the baseline w/o HPV curve, and the w/ HPV RBC transit times distributions are modestly homogenized and right shifted. Moreover, in response to a critique from another reviewer, we added the change in total resistance to **Table 5**. We can see that both UVC and HPV similarly increase total resistance and decrease cardiac output. However, we see a greater change in the oxygen uptake from HPV compared to UVC. This difference in oxygenation, despite similar total resistance and cardiac output, is due to the differences in the distribution of blood flow. To look at a single number, this is crudely summarized the by the flow coefficient of variation which is also reported in Table 5.

We have updated the Discussion section to report this:

Lines 601 - 605

This is a model with many components and it is difficult to understand exactly what is necessary. You need to provide a justification of the inclusion of each component, and a thorough analysis of the sensitivity of the model to each component and parameter. There is no evaluation of the contribution of the time-varying alveolar pressure, nor the dynamic model of perfusion. The introduction suggests that these are important factors, but no results are provided that show why they are important. Similarly, there are many tunable parameters in the model. It is essential that you provide a thorough sensitivity analysis to determine their contribution to the model behavior.

We thank you for this criticism. We have performed a sensitivity analysis model on parameters, and have reported the results in the Model Parameterization and Discussion sections

Lines 360-379, 676-691, Table 4

A much simpler model with very basic rules could be used to reach the same conclusions. That is, a model that has a simple change in dimension of vessels (increased resistance) in proportion to the downstream alveolar PO2 would give the same conclusions as your model under your scenarios. You therefore need to focus on exactly what is novel in your model, and what new knowledge can be derived from it.

We respectfully disagree. A simpler model, like you describe, would indeed show that increasing vascular resistance would decrease blood flow. Decreased blood flow would reduce RBC transit time, and the reduced RBC transit time would improve oxygenation. However, the situation is more nuanced in a network of gas exchange units. Increasing the resistance to a single gas exchange unit in the network will decrease flow through that gas exchange unit, BUT, since flow is conserved, the other gas exchange units in the network will experience an increase in flow. How does the change in resistance upstream of one unit affect the oxygenation throughout the whole network? Moreover, what happens if some gas exchange units have their upstream resistances increasing and others have them decreasing? To address these questions, a more complex model is needed.

We agree that we need to be clearer on what is novel in our model and recognize that our lack of clarity may be source of our disagreement. We have updated our Methods and Discussion sections to clarify the novel components of our model and what the contributions of this study are.

Lines 90-97; 463-477

Specific comments

Abstract - “to redirect blood flow to areas”: it is more correct to say “to direct blood flow away from areas that have low PO2”. And same elsewhere throughout the manuscript.

Older literature has explained HPV as a mechanism that “redirects blood flow to other areas,” however we do agree that it is more accurate and mechanistic to say that HPV “directs blood flow away from areas that have low PO2.”

We have updated the abstract and the entire manuscript to reflect this phrasing

Author summary - Most pulmonary embolism does not result in decreased blood oxygen.

We respectfully disagree. For example, Bĕlohlávek et al. (2013) state that “Arterial or venous blood sampling for blood gas analysis is a routine laboratory test. A typical picture of acute PE is a combination of hypoxemia and hypocapnia with respiratory alkalosis as a result of compensatory hyperventilation. Rarely, in severe forms of PE, hypercapnia can be present. Blood gas analysis in PE patients enable more accurate risk stratification of an individual patient, primarily in terms of development of organ dysfunction.” Perhaps the reviewer is referring to chronic PE? This may be the source of our disagreement, as patients with chronic PE can be asymptomatic and challenging to diagnose.

We have updated our Author Summary to refer to acute PE.

Bĕlohlávek J, Dytrych V, Linhart A. Pulmonary embolism, part I: Epidemiology, risk factors and risk stratification, pathophysiology, clinical presentation, diagnosis and nonthrombotic pulmonary embolism. Experimental & Clinical Cardiology. 2013;18(2):129.

You need to be clear that HPV is an active regulatory mechanism. It is not the primary mechanism for V-Q matching (gravity is, which you mention later for large animals).

We agree that we need to be clear that HPV is an active regulatory mechanism

Lines 24-25, 474-477

Introduction – this is overly long and much of it is superfluous to the actual study. e.g. everything about West’s zones.

We agree. We have significantly reduced our introduction to be more focused and clearer.

Lines 52-57 – but is any of this considered in the model?

With respect to the previous comment, we have removed this section from the introduction.

Line 19 – blood does not ‘pool’ in the lower lung. That implies that it is stationary.

We have edited this sentence to now say “resulting in increased blood flow through the lowest part of the organ”

Lines 19-20

Line 19 – the marginally higher blood volume in the lower lung does not ‘compress’ it. Tissue compression is a consequence of the weight of the entire lung tissue, which includes blood and extravascular fluid.

Thank you for this correction. We have corrected our language

Lines 20-21

Line 23 – there has been a large body of experimental work to understand how V-Q matching affects gas exchange in normal and pathological conditions so the construction of the sentence at Line 22-23 is not correct.

Thank you for pointing this out. This sentence does not accurately convey our thoughts. There has certainly been a tremendous amount of effort put into studying V/Q matching. Our intent was to communicate that there is a gap in field’s understanding of how active regulatory mechanism (such as HPV) regulate gas exchange.

Lines 24-25

Line 29 – experimental studies have suggested that hypocapnic bronchoconstriction could be an important regulatory mechanism. This has not been proven, but there is some evidence.

This is a fascinating point - we have added this into our Introduction.

Lines 29-32

Line 42 – what do you mean by the ‘alveolar-capillary boundary’? Are you actually referring to the entire alveolar-capillary interface? Or to the smallest arterioles that supply the alveoli?

We are referring to the smallest arterioles that supply blood to the alveoli. We have edited these sentences to improve clarity.

Lines 46-47

Line 107 – and later, throughout: ‘Validated’ is not correct.

We have changed “validated” to “calibrated” throughout the revised manuscript

Line 144 – are the Fahraeus and Fahraeus-Lindqvist effects relevant to the size of your model vessels?

The smallest Strahler order of blood vessels in our model are approximately 185 um, and the largest Strahler order of blood vessel are approximately 600 um. When we plot the vessel diameters along the curve defined by Preis et al.’s nonlinear viscosity law (Pries A, Secomb T, Gessner T, Sperandio M, Gross J, Gaehtgens P. Resistance to blood flow in microvessels in vivo. Circulation research. 1994;75(5):904{915.), we can see the Fahraeues-Lindqvist effect (decrease in viscosity as the vessel diameter decreases) is relevant, but the Fahraeus effect (increase in viscosity as tube diameter approaches that of a RBC) is not.

We are able to generated more detailed networks that do have diameters in the regime where the Fahraeus effects was relevant, however we chose to use less detailed networks as a compromise to balance the computational expense of the solving the system ODEs.

Line 155 – How were the networks manually calibrated, and how much calibration was required?

We manually calibrate our network anatomy to the morphometric data by adjusting the size and shape of the finite-element meshes generated by Polymesher, and manually adjusting the value of k (hydraulic conductivity per unit length).

To our knowledge, hydraulic conductivity per unit length is not a quantity routinely measured or reported in the literature. Thus, we treated k as an adjustable parameter and adjusted it to find a high quality fit to the data in Jiang et al (Jiang Z, Kassab G, Fung Y. Diameter-defined Strahler system and connectivity matrix of the pulmonary arterial tree. Journal of Applied Physiology.1994;76(2):882–892.). The manual calibration required to find a satisfactory value for k was minimal. We primarily varied it by orders of magnitude. It is possible to optimize k via a parameter estimation procedure (ex: non-linear least squares), but the improvement in the quality of fit would likely be minimal - especially given the inherent limitation of our generated networks being 2D compared to true lungs being 3D.

We have added these sentences to improve clarity:

Lines 140-141; 268-272; 490-497

Equation 2 – presumably Pv is the transmural pressure for the vessel (internal minus external pressure), where the external pressure is taken as Palv? You need to define this.

You are correct. We have clarified these definitions.

Lines 149-152

Equation 3 – What exactly is Palv, how is it defined, and does it change during the simulation? If this is a dynamic model then I assume that it must change. (Edit – yes, but I only see that because an amplitude is given in Table 1). How does the fluctuation in Palv relate to Equation 12?

Thank you for pointing this out. Palv is the physical pressure (mmHg) within the alveoli. Your intuition that Palv is related Equation 12 is correct. We have assumed that Palv is sine wave similar to Qalv. We have added these details

Lines 191-192; Equations 12-13

Line 182 – how are the capillary segments defined? Do you use a sheet-flow model for the pulmonary microcirculation, or do you assume a simple capillary branch? It seems from Line 212 that you are treating a single ‘capillary vessel segment’ as a whole gas exchange unit (i.e. representing many capillaries with a large surface area). You must provide an explanation and justification in the methods.

You are correct, we are treating a single capillary vessel segment as a whole gas exchange unit. We are aware of sheet-flow models (Fung YC, Sobin SS. Theory of sheet flow in lung alveoli. Journal of Applied Physiology. 1969 Apr;26(4):472-88.), however we have chosen to use this representation primarily out of the reduced computational cost. We have added a more thorough explanation of justification of this choice.

Lines 173-177; 544-553

Lines 215 and elsewhere – you should refer to oxygen content, not concentration.

We understand the reviewer’s point. However, while “Concentration” is a quantity with units (mol/L), “Content” may be ambiguous and could be interpreted as concentration, mass, or partial pressure.

We have addressed this issue by clarifying in the text that the variable C_O2 is the total (bound plus freely dissolved) oxygen in the blood.

We also identified a typo where we wrote “oxygen mass” but meant to write “oxygen concentration,” and have made this change (Line 213).

Line 256 – As mentioned above, you need to provide explanation of how much manual calibration was required to achieve this.

We agree.

Lines 140-141; 268-272; 490-497

Line 271 – I appreciate the effort put in to comparing or calibrating to experimental data. But it is not appropriate to call this a “Model validation”. You have introduced a global scaling parameter (T*) to allow reasonable match for one set of studies, and then you optimize other model parameters to provide a good fit to other data. So this is a calibration to provide a reasonable baseline model behavior, rather than a ‘validation’. As such, some of this should be in your methods and not results. Validation requires that you compare against independent data (not used for fitting/calibration), and ideally this should be testing an emergent behavior.

We have reorganized the paper by moving the contents of the “Model Validation” subsection under the “Model Parameterization” subsection in the Methods

Line 291 – Here you introduce T*. Your pressure-flow relationships are presumably sensitive to the resistance of the capillary bed, but you have not given any information about its dimensions or behavior. The different experiments could also cause different patterns or amounts of capillary bed recruitment. However, by using T* you have assumed that any difference between model and data is purely related to tone, but other factors could be at least as important. Please explain and justify.

Thank for pointing this out. We have added details regarding capillary segment parameterization to the Model Parameterization: Vascular Mechanics subsection of the Methods

Lines 285-300

Capillary recruitment is a phenomenon we are deeply interested, however accurate and robust representation of it in our model exceeds the scope of this study. To mitigate this uncertainty, we chose studies that performed their experiments under Zone 3 conditions (alveolar pressure < venular pressure) as a strategy to control for differences in capillary recruitment patterns. We have added a few sentences in the Discussion to more completely discuss the limitations of our model and how we are looking forward to modeling capillary recruitment.

Lines 701-710

I can’t tell whether the simulations were run for normoxia or hypoxia. HPV has been shown to be not important under normoxic conditions, but Equation 22 suggests that you include the effect across all partial pressures. Please explain and justify.

The simulations presented in Figures 6-8 we run under normoxic conditions. And you are correct, equation 22 does apply over all partial pressures.

We are aware of the studies which suggest that HPV is not important under normoxic conditions (Arai TJ, Henderson AC, Dubowitz DJ, Levin DL, Friedman PJ, Buxton RB, Prisk GK, Hopkins SR. Hypoxic pulmonary vasoconstriction does not contribute to pulmonary blood flow heterogeneity in normoxia in normal supine humans. Journal of Applied Physiology. 2009 Apr;106(4):1057-64). However, we believe there is more nuance to the conclusions of this study. The hypothesis of this study was that mild hyperoxia would inhibit HPV and decrease the heterogeneity of blood flow. Their results show that under mild hyperoxia that there was little change in the distribution of blood flow compared to normoxic conditions. We are skeptical of hyperoxia exposure being an effective perturbation to uniformly inhibit HPV. Under normoxic conditions there is heterogeneity in the distribution of airflow and oxygen in the airway, meaning that there are likely some regions of the airway that have low amounts of oxygen and HPV could be activated. Under hyperoxic conditions, the average oxygen content in the airway certainly increases, however the heterogeneity of airway oxygen distribution is likely unchanged (Starr IR, Lamm WJ, Neradilek B, Polissar N, Glenny RW, Hlastala MP. Regional hypoxic pulmonary vasoconstriction in prone pigs. Journal of Applied Physiology. 2005 Jul;99(1):363-70). Thus, there may still be regions of the lung ventilated with small amounts of oxygen. Our criticisms of the Arai study aside, we do agree that the physiological benefit of HPV is likely of significant relevance during hypoxia exposure, conditions where oxygen consumption is increased (cardiovascular exercise), or when there are significant structural occlusions of the vasculature or airway (pulmonary embolism, pulmonary fibrosis). We have added additional simulations with different fractions of inspired oxygen to the revised manuscript.

Lines 426-439; Figure 9

To improve clarity, we made edits to the Methods section to justify the functional form of our empirical HPV model and to the Discussion section to state our opinion on the limitations of the Arai study.

Lines 231-234; 238-245; 247-256; 636-661

Line 357 – if the mean transit time increases, this implies that the cardiac output has decreased or the blood volume of the model has increased. I assume that you compared cases where the former was equal, so did you have sufficiently different blood volume in the different scenarios to give these different mean transit times? Edit – I have now re-read the manuscript and can see in Table 4 that your total flow decreases in HPV because you use pressure boundary conditions. So of course your transit time will be longer.

Important – can you please explain how your cardiac output can be reduced to approximately one third yet the total oxygen flux increases? (network A and B without and with HPV).

This is an excellent point, and in line with a previous comment of yours. If cardiac output is reduced, the mean RBC transit time will increase. With an increased mean transit time, RBCs take longer to traverse the pulmonary circulation, and they have more opportunity to interact with the oxygen in the alveoli and uptake more oxygen. Thus, in the absence of any regulatory process, we expect a decrease in cardiac output to increase oxygen uptake.

To make comparisons between the w/o HPV, w/ HPV simulations, and UVC simulation, we report the normalized RBC transit times. On the axis is the transit time divided by the mean transit time. We mentioned in a response to one your previous comments that there was mistake in how we plotted our RBC transit times, which likely contributed to the need for you to make this comment.

To improve clarity, we added more details to our Discussion sections.

Lines 463-470; 601-605

Line 358 – this is only correct if the increased transit time occurs within the gas exchange (your capillary) region. But I believe that in your model the increased transit time is in the larger vessels. Unless I am missing something about the behavior of the capillary in your model (which is not described in the methods).

For there to be a change in the transit time through the capillary vessels, there must be a change in the transit time of the upstream larger vessels. The increased transit time is in the larger vessels. This increases the transit time through the capillary vessels.

Line 365 – how can there be more high air flow regions when you have set the air flow to be the same everywhere?

Thank for pointing this out. In a true lung, regions with a large V/Q ratio could be due to high air flow, or small blood flow. In the case of our simulation results, this is due a small blood flow. We have edited this sentence to be more clear.

Line 415

It looks as though you have used P50 for dog. I assume that this is more similar to human than rat, but it is worth commenting on this in the discussion.

Thank you for pointing this out. The P50 and Hill coefficient (n) we originally reported were for dog, however this was a mistake. When assembling the LaTeX, we copied the formatting from a previous manuscript the hemoglobin saturation parameters for a dog were left unedited. We have updated our table to reflect that we are using P50 and Hill coefficient (n) for a rat.

Line 465- I strongly disagree that the model has been ‘validated’. It has been calibrated to existing data. And it does not match some important data (e.g. RBC transit time).

As stated in a previous response, we have revised the manuscript to replace the language of “validated” with “calibrated.”

In a response to one your previous comments, we mentioned that there was mistake in how we plotted our RBC transit times. We have corrected these details, and removed these erroneous comments from our discussion.

To improve clarity, we have also added more detail to our Discussion section.

Lines 471-477; 601-605

**Answer to Reviewer #3**

Authors propose a novel computational model of ventilation-perfusion matching in pulmonary circulation. Work couples three different models- one related to vascular mechanics, one related to oxygen transport and one related to vasoconstriction. It is able to capture data from literature, quantitatively in some and qualitatively in some other. The coupled model is able to show that the hypoxic vasoconstriction homogenizes regional blood flow and alv-capillary oxygen flux.

Strength of the paper is it has elegant ideas related to coupling models and has captured some results in literature successfully. The weakness is the methods section lacks details to understand the model completely.The paper will be stronger with some more rigor on parameter estimation. Comments below:
-- Systematic sensitivity analysis of generated distributed networks to user inputs is missing.

We thank you for pointing this out. Another reviewer has also requested a sensitivity analysis and it has been added to the revised manuscript

Lines 360-379; Table 4

-- There is a scale bar on the distributed networks (Figure 2 and 3). If the networks are non dimensionalized, utility and findings can be extended across species. Can the authors attempt/ give their thoughts on non-dimensionalizing the model?

Generating the structure of networks using our algorithm is generalizable to any size. The operative challenge is assigning a radius to each vessel segment. We did this by using fluid flow through the network as the governing condition, and assuming of a constant hydraulic conductivity per unit length (k). This is a potentially limiting assumption which we have discuss in the revised manuscript

Lines 140-141; 490-497

We did non-dimensionalize the vascular mechanics ODE early in model development, along with a linear stability analysis. Both analyses were performed with a version of the model that assumed constant viscosity. Results from both were underwhelming. For a given vessel segment, the vessel wall pressure (Pv) and inlet flow rate (qin) are the variables, and inlet pressure (Pin) and outlet flow rate (qout) act as boundary conditions (we do not mean boundary conditions as if this were a PDE, but functions that define the “boundaries” of the segment). The linear stability analysis showed us that qin stably approaches qout, and Pv stably approaches Pin for all parameter values. Non-dimensionalization did not reveal any latent features of model behavior and dynamics. In retrospect, this was obvious as the ODEs are a linear system of equations. The only notable thing we learned from this analysis is that some parameters (ex: elastic modulus of vessel – E) affect the time scale it takes for the model to relax to steady state.

These analyses are limited by the assumption of constant viscosity when blood is a non-Newtonian fluid. We account for rheological properties via Preis et al.’s non-linear viscosity law (Pries A, Secomb T, Gessner T, Sperandio M, Gross J, Gaehtgens P. Resistance to blood flow in microvessels in vivo. Circulation research. 1994;75(5):904{915). Having the viscosity depend on the radius of the vessel would certainly complicate the non-dimensionalization. We chose to not pursue this with our ODE vessel mechanics model, as this analysis would be more appropriate with a PDE formulation based on the Cauchy momentum equation, or Navier-Stokes equations with a corresponding constitutive relationship for viscosity.

-- Please include more details on 'manual calibration of network anatomy'. What was used and changed for calibration? How hard was 'manual calibration'? Is it repeatable across users?

This was brought up by a previous comment.

Lines 140-141; 490-497

-- Airway-vascular oxygen transport section has no references - was the model formulated in this paper? Or is it relying on prior work? This section is hard to to conceptualize and understand with the description provided. Can the authors include more schematics to complement transport equations and descriptions? I am unable to tell airway 1 and airway 2 in Figure 1 (also why "" on "airway 1"?)

The oxygen transport model was formulated in this paper. In general, the approach we used is was a compartmental mass-balance. We added a few sentences to clarify the form of the equations.

Lines 181-186; 191-192; 209-210

We have also added a Figure to the Appendix to illustrate the structure of the airway model.

Lines 721-723; Figure A1

-- The formulation of airway-vascular oxygen transport is confusing - the governing equations are in partial pressures, mass of O2 in (moles) (M) and concentration of oxygen. Also, C_o2 is described to be mass of oxygen(mol) in paragraph but is described to be concentration of oxygen prior to equation 20. Can the authors reformulate the equations to keep descriptions consistent through the model?

We thank the reviewer for identifying these inconsistencies. We identified a few critical typos (ex: C_O2 is a concentrations) which may have been a major source of your confusion and have corrected them in the revised manuscript.

Lines 198, 206, 213

--Motivation for functional forms used in 'Empirical Hypoxic Pulmonary vasoconstriction' is missing.

Thank you for pointing this out. We have added an explanation to our Methods section describing the basis of the functional form for out empirical HPV model.

Lines 231-234; 238-245; 247-256

--Why is the signal generated by oxygen content exponential - is there biological data to motivate this form?

There is biological data demonstrating the response of HPV at various fractions of inspired oxygen (Hlastala MP, Lamm WJ, Karp A, Polissar NL, Starr IR, Glenny RW. Spatial distribution of hypoxic pulmonary vasoconstriction in the supine pig. Journal of Applied Physiology. 2004 May;96(5):1589-99.). However, there is not robust data available demonstrating how those responses map to a molecular signal (ex: membrane potential as a function of oxygen). This remains is an open question we are deeply interested in. We reasoned that any function that has signal increase when the oxygen content decreases would be appropriate starting point. The exponential form was chosen primarily out of simplicity (ex: smooth curve with no discontinuities, strictly positive). The edits we made in response to your previous comment address this point.

--Is there evidence of spatial decay of transduced signal in vivo?

Yes, landmark studies by Segal et al. (Segal SS, Welsh DG, Kurjiaka DT. Spread of vasodilatation and vasoconstriction along feed arteries and arterioles of hamster skeletal muscle. The Journal of physiology. 1999 Apr 1;516(Pt 1):283.) has demonstrated this. It is worth nothing that there are very few studies on conducted vascular responses in the pulmonary vasculature. Work by Wolfgang Keubler’s research group (Wang L, Yin J, Nickles HT, Ranke H, Tabuchi A, Hoffmann J, Tabeling C, Barbosa-Sicard E, Chanson M, Kwak BR, Shin HS. Hypoxic pulmonary vasoconstriction requires connexin 40–mediated endothelial signal conduction. The Journal of clinical investigation. 2012 Nov 1;122(11):4218-30.) has been the primary motivation behind our empirical HPV model. The edits we made in response to your previous comment address this point.

--What does the time constant \tau physiologically represent in equation 24?

Tau represents the rate at which vasomotion occurs. In other words, how quickly it takes for a vasoconstriction to occur in response to hypoxia. It is worth nothing that is nuanced point regarding HPV. Experimental studies have shown that there is an acute response observed within 10-20 minutes of hypoxia exposure, and a modest but prolonged response over the course of multiple hours. Our model only captures the time course of the acute response.

--Equation 25 and 26 are elegant ways to couple lumped parameters and vascular tone. Add details so a naive reader understands why you intuited this functional form. Also, is value of T_j bounded for in vivo conditions? -Elaborate

We thank you for your compliment. T_j is bounded by the parameters T_Max and T_min. We have added a more detailed description in our Methods section to explain how these equations couple vascular tone to lumped parameters.

Lines 249-250; 253-256

-- Overall, the novel aspects of the 'Methods' are not clear. Three different models are presented -- one related to vascular mechanics, one related to oxygen transport and one related to vasoconstriction -- which sections of these models are novel? Or is it the coupling thats novel? Clarify in methods and add a paragraph in discussion.

Thank you for pointing this out. Our empirical HPV model and the coupling of the three models are primary our novel contributions. The mechanics model was originally done by Safaei et al. (Safaei S, Blanco PJ, Müller LO, Hellevik LR, Hunter PJ. Bond graph model of cerebral circulation: toward clinically feasible systemic blood flow simulations. Frontiers in physiology. 2018 Mar 2;9:148.). As stated in a previous response, we did derive the oxygen transport equations ourselves on the basis of compartmental mass-balance. We have added a few sentences to the Method and Discussion sections to make our contributions more clear.

Lines 90-97; 463-477

-- The coupling with vasoconstriction and vascular mechanics is clear but the coupling between airway and vascular oxygen transport is unclear -- can you add a separate section/figure talking about coupling and what parameters/quantities are exchanged between these models?

We believe that some of our typos in the Airway and Vascular oxygen transport section partly why this comment was made. We hope the edits offer some clarification

Lines 181-186; 191-192; 209-210

We also note the HPV model does not directly interface with the oxygen transport equations (other than in generating and HPV signal from oxygen in the alveoli). The HPV model regulates blood flow, and the change in blood flow is what is altering oxygenation.

--Is the assumption of constant hydraulic conductivity/length through the vascular tree valid in the pulmonary tree? Especially given the heterogeneity in composition and function through the tree. Also, the reference for the value is missing in text.

This is an excellent point. We have not provided a reference for this value, as we are not aware of one in the literature. Adjusting this value by hands is how adjusted the morphometry of our networks to calibrate it to data from Jiang et al (Jiang ZL, Kassab GS, Fung YC. Diameter-defined Strahler system and connectivity matrix of the pulmonary arterial tree. Journal of Applied Physiology. 1994 Feb 1;76(2):882-92). We have added a section to our Discussion to unpack the validity and potential limitations of this assumption.

Lines 140-141; 268-272; 490-497

--In figure 4 - the fit for Network B is not as good as Network A and C. The type of network is also affecting the differences between 'w/o HPV, w/HPV and UVC' across Figure 6,7 and 8. Does this reflect model's ability to capture only a certain kind of network architecture well?

Network B is mechanically stiffer than the other two networks, which is likely explained by there being bifurcation early in the vascular tree. The result of this there being effectively 2 distinct networks. We believe that is a short coming of how we assign radii to vessel segments in the network generation step. How network architecture influences mechanics and oxygen transport is a fascinating topic but exceeds the scope of our study. We have added a section to our Discussion to better address the limitations of our generated networks.

Lines 507-511

--Figure 5 and Table 2 -- the fits for hypoxic conditions are off by 15-20%, does this mean the model struggles to capture hypoxic conditions? Why? Is there a way to fix it? Also, acknowledge these shortcomings in Discussion.

Thank for pointing this out. This is a result of two interacting phenomena. (1) We do not account for physiological changes in systemic oxygen consumption under hypoxic conditions. In other words, we assume that the oxygen concentration entering the pulmonary circulation is the same under normoxic and hypoxic conditions (our model does not have a closed loop of circulation). (2) How we account for oxygen bound the hemoglobin and freely dissolved in plasma. Even under hypoxic conditions, the model is still operating on the relatively plateaued potion on the oxy-hemoglobin dissociation curve above the half-max saturation. In this region of the curve, it takes a large change in oxygen concentration to see a change in oxygen tension.

Of these phenomena, we suspect (1) is the primary culprit in our model’s struggle to capture hypoxic conditions. Presumably, this could be improved by developing a model that has a closed loop of circulation and physiological oxygen consumption. Unfortunately, this is a not a trivial task as there are many local and whole-body responses that control oxygen consumption and exceeds the scope of what we wanted to accomplish with this study. We have added a section to our discussion to address this.

Lines 554-565

--The model has a lot of parameters and very few measurements, can the authors comment on the identifiability of the system? Also, in Table 3 if Tmax and Tmin are correlated does it make sense to estimate only one of them?

T_Max and T_min represent the bounds of the vascular tone in our empirical HPV model. There is no data available to inform how we set these parameters, so they are inherent sources of uncertainty. From the standpoint of structural identifiability, we would certainly be ideal to keep one fixed and estimate the other. However, this becomes a circular problem of not knowing what to keep one parameter fixed at while we estimate the other. This issue is what ultimately led us to using BELUGA, a genetic algorithm, so could estimate both of these parameters, rather than a gradient-based method (ex: Levenberg-Marquardt) which are much more susceptible to numerical instability and/or non-reproducible results from parameter identifiability. We have added a section to our Discussion to unpack this rationale.

Lines 662-676

-- Given the comment in discussion - have the authors looked at RBC transit time as objective function in their estimation? Its physiologically motivated so makes a great candidate for objective function.

We realized from a comment from another reviewer that there was a bug in our code in how we were plotting RBC transit time distributions (we were not properly normalizing them). By correcting this, our RBC transit times now do align very well with those reported in the literature, so we have removed this comment from the Discussion.

The suggestion to use RBC transit times in our objective is interesting one, and an option we considered. We were unfortunately able to find data in the literature where RBC transit times were measuring under normoxic and hypoxic conditions. This did however turn out to be a benefit, as we have used our model to predict how HPV affects RBC transit times. If RBC transit times were used in the objective function, then these “predictions” would just recapitulate the data we fit the model to.

Minor:
--Problem with formatting of Table 1- resize it to avoid overlap with page header and footer

We agree that this is an issue, however we are not sure how to correct this without fundamentally altering PLOS Computational Biology’s LaTeX style file. Assuming this study is accepted, we hope to work with a copy editor to ensure this table is clearly presented.

-- thickness(h) in Table 1 is in mmHg?

Thank you for pointing this out. We have corrected this.

--The resistance in lumped parameter network is for viscous dissipation - manuscript has used phrases like 'viscous damping', 'viscoelastic damping' etc. -please change it and use these terms carefully.

Thank you for point this out. We have corrected this.

--line 337 - as 'shown' in Figs 6A, 7A, 8A ....

Thank you for point this out. We have corrected this.

--line 341 -- check figure numbers for '6D, 7D and 7D'?

Thank you for point this out. We have corrected this.
