# Supplementary material for: Hypoxic pulmonary vasoconstriction as a regulator of alveolar-capillary oxygen flux: A computational model of ventilation-perfusion matching
Source: PLoS Comput Biol. 2021 May 6;17(5):e1008861. doi: 10.1371/journal.pcbi.1008861 (PMC8130924; doi:10.1371/journal.pcbi.1008861)
Supplement: Attachment — Submitted filename: responses_to_reviewer_comments2.docx [file pcbi.1008861.s008.docx]

**Answers to Reviewer #1**

Reviewer #1: The authors are to be commended for the many changes and improvements (including additional simulations) made to the manuscript in response to the submitted reviews. However, in the opinion of this reviewer, addressing the following issues could improve the paper further:

MAJOR COMMENTS

1. Abstract needs to clearly state that 2D network models are used to represent the vascular and airway structures of the lung, and that capillaries are not explicitly represented

We agree – thank you for this suggestion

2. LL527-534: Authors should reconcile the assumption of fixed Hct with the calculation of vessel radii from Eq 1 using viscosities that are a function of Hct

Thank you for this suggestion. We have added this reconciliation to our revised manuscript (L577-582).

3. Discussion: Please include a section on the limitations of using fixed pressure boundary conditions for calculations of flow, resistance and oxygen transport as presented in Table 5, including a discussion of how decreased overall flow can improve oxygen flux

Thank you for this suggestion, we have added a few sentences to the discussion of our revised manuscript (L652-657). Our model assumes constant physiological values for pulmonary arterial pressure and left atrial pressure. This assumption may not be valid under conditions such as pulmonary hypertension or exercise. Yet, the assumption allows us to investigate how the pulmonary vasculature redirects flow with a constant driving force. In other words, it is an *advantage* of the simulation experiment that we can hold all other things constant and modulate one variable at a time.

MINOR COMMENTS

- Please clarify (in proof) that Fig A1 refers to Figure 11 (and Fig A2 to Fig 12)

Thank you for pointing this out. This should be addressed during the copy-editing process. We are not aware of a way through PLOS’s submission portal to label figures as appendix figures.

- Captions for figures 6, 7, 8, 10 do not indicate dimensions of the scale bar

We have added this detail to the caption for these figures.

- L331: Hypoxic air fraction still reads 21%

We have corrected this typographical error

- L726: For clarity, please explicitly state proportionality constant for tidal volume in Figure A2/12 relative to Q*_V (ml/s)

We have updated L771 to report that the proportionality constant for tidal volume (TV) and Q*_V is k = 0.272 = TV/Q*_V

- LL601-605: Please include in Fig 5 caption that T* = 0.8 was used to simulate UVC for all three networks

We believe you mean Figures 6,7, and 8? We agree and have added these details to those figure captions.

- Table 1: What are the nominal values of Q*_P and Q*_V? Is [46] the correct reference for rat values? On what basis (or reference) was a value of 115 mmHg chosen for P_HPV?

Q*_P = 0.655 and Q*_V = 2.9702 – we have updated Table 1 to report these values, and have added a few sentences to the “Model Parameterization” section to report these details (L367-372). [46] is the correct reference – while this is a study using human subjects, we rescaled the human cardiac output and ventilation measurement to reflect that of an adult rat. To make this clearer, we have added details to Table 1 showing how we rescale these measurements in the “Estimator” column.

We have updated Table 1 to report an Estimator for how we set P_HPV and added an explanation to the text (L376-381).

- Presumably copyediting will resolve the numerous spelling and typographical errors that remain and/or have been introduced (e.g. LL23, 381, 385, 437, 453, 530, 534, 535, 570, 602, 606, 613, 618, 619, 621, 651, 658, 662, 673, 689, 704, 710)

We sincerely appreciate your patience with these typographical errors. We have corrected those that you have identified and have spent a considerable amount of time combing through to the manuscript in hopeful preparation for a smooth copyediting process.

**Answers to Reviewer #2**

Reviewer #2: The authors have made a good effort to respond to my questions but have not taken on board my most significant concerns. There are additional issues that need to be addressed in light of the response to some of my questions.

From my original review:

• “Please interpret your results in the context of intact lung physiology”. The primary conclusion (that HPV homogenizes Q) is only relevant to a uniform V, which is not a physiological condition. Either the language needs to change to be clear that this is an artificial test condition, or the authors need to introduce a realistic baseline V (for rat this just needs to have heterogeneity). The authors have run yet more simulations in response to this major concern rather than address it directly. Under normal physiological conditions V is not uniform. So it is entirely misleading to say that HPV ‘homogenizes’ Q. This has not been addressed at all in the responses. In fact, the new results raise a further issue: that under the exact conditions where we expect that HPV will be important the model does not predict a realistic response. You must go through the manuscript thoroughly and very clearly point out that you are considering an artificial situation to test your model. The general non-respiratory reader will not be aware that V is not uniform, so where you say in the abstract “with a uniform ventilation distribution” it is likely to be interpreted as physiological.

Thank for taking the time review our study. We agree that a non-respiratory reader could mistakenly assume true ventilation distributions are uniform. To this end, we have edited the abstract and manuscript to be clear that a uniform ventilation distribution is an artificial test condition and affects the interpretation of our results. Moreover, we have revised our wording to say that “HPV matches perfusion to ventilation” rather than “HPV homogenizes perfusion.”

• “You need to be clear that HPV is an active regulatory mechanism”. The manuscript still implies that HPV is the primary mechanism regulating V/Q matching. Please fix this. This is throughout the manuscript starting from the abstract (e.g. “model of ventilation-perfusion matching” needs to be modified so the reader understands this is a model of V/Q matching via HPV only).

We agree. We have gone through the manuscript to make clear that we are presenting a model of V/Q matching through HPV only.

New concerns arising from responses:

• It is now clear (but only because I asked) that HPV is assumed to be active in normoxia. This goes counter to the current literature. I am willing to be convinced that HPV is more significant in normoxia than experimental studies suggest. But I am concerned that you have started with this opinion and therefore built this assumption into your study without 1. being very upfront and clear that this is the case, and 2. thoroughly testing the assumption. What would happen if you started with a ventilation-perfusion distribution that was heterogeneous but quite well matched (as per the normal lung)?

Thank you for pointing this out. We have edited our introduction by moving the “Physiological relevance of hypoxic pulmonary vasoconstriction” section in the discussion to the introduction (L56-86), and state in L263-264 that we have built the assumption of HPV sensing oxygen during hyperoxia/normoxia into our model.

Your second point about starting with a ventilation and perfusion distributions that are quite well matched is quite interesting, but this raises a challenge of how we specify these distributions *a priori*. While the distribution of air flow is heterogeneous it is not random, and the structure of this heterogeneity is almost certainly a consequence of the airway branching structure. Furthermore, what is the explanation of why ventilation and perfusion are well matched? The answer to this question is currently contentious, however the explanation is some combination of the interwoven nature of the airway and vascular anatomies, passive phenomena such as the influence of gravity, and physiological regulatory mechanisms (such as HPV). Simply put, the only way to start with V/Q distributions that are well matched is to put feedback into the model that makes them well matched. The million-dollar question that the field at large is still grappling with is how to disentangle the anatomical and physiological regulatory components that govern V/Q matching. We have added the above comments to our Discussion (L671-679, L681-682).

• How much vascular constriction actually occurs for different PO2 levels? We are not actually shown any results for PO2, only O2 uptake.

We have reported this information in a Fig A3 in the Appendix.

• Now that I have clarity on the model being sensitive in normoxia, can you please provide results on the distribution of PO2 (as per the maps in Figures 6,7,8).

Absolutely – we have added this information as a figure (Fig A3) to the Appendix.

• A question that occurs to me in response to the issue on normoxia: if the model adapts to homogenize Q towards the uniform V, presumably the PO2 actually homogenizes. So what sustains the HPV response if PO2 becomes close to ‘normal’?

Your intuition is correct, the alveolar PO2 is homogenized by HPV. We think of HPV as a physiological feedback mechanism, where the function of the mechanism is to homogenize the driving force (alveolar-arterial O2 difference) underlying O2 uptake by matching the distribution of Q to V. In engineering terms, HPV can be thought of as a control system that regulates O2 uptake by controlling blood flow distribution. When the alveolar-arterial O2 difference distribution deviates from homogeneity, we see the effect of HPV. How well HPV can re-homogenize the alveolar-arterial O2 difference is context dependent.

Regarding your question “what sustains the HPV response if PO2 becomes close to ‘normal’?” - the observed HPV response will be relative to what we consider baseline and what we consider a perturbation. We present a situation in our study comparing lungs with and without HPV. This situation is purely theoretical as there is not an experimental or clinical analogue as the field is currently unable to directly inhibit molecular and physiologic integrators of HPV. Our model simulations suggest the distribution of Q, and V/Q ratios observed in healthy individuals is (at least in some part) due the continual action of HPV.

• When regions of zero V are introduced the model gives a modest change in Q. But this is the condition in which we expect to see HPV being important. You have mentioned this in the limitations, but this is creating a rather backwards story: HPV is the lung’s primary regulatory mechanism to divert flow when there is an abnormality, so we should expect the model to perform well in this situation. Normoxia in a ‘normal’ lung should be the additional scenario.

As highlighted by the modest change in Q in response to regions of zero V, we believe our empirical model of HPV does not fully capture the true function of this mechanism. In Fig A4 we show the HPV tone (T) from HPV for each vessel segment in the model. One can appreciate the largest HPV T tend to be in the distal segments and near regions with zero V, whereas the more proximal segments tend to have lower T. This is explained by the mathematical form (exponential decay) of how we assume these signals are conducted upstream through vessel walls.

• Original: “Important – can you please explain how your cardiac output can be reduced to approximately one third yet the total oxygen flux increases? (network A and B without and with HPV).” In response, the authors say that the reduced cardiac output means a longer transit time which in turn means more time for oxygen uptake. But this is only correct for transit times that are less than the time required to reach saturation. This is about 0.2 seconds. For the rest of the transit time the RBCs are not taking up oxygen. Can you please explain how this works in your model. Do you include (or see) saturation? Your model seems to contradict a flow-limited system (where increased flow should increase the net oxygen uptake).

We have verified that the model equations and associated computer codes are mass-, and volume-conserving, and thus do not contradict any principles of mass transport.

The increase in oxygen flux with decreasing flow is a perhaps non-intuitive result. The reason for this behavior (summarized in Table 5) is that without any mechanism to achieve V/Q matching, blood flows throughout the network are highly non-physiologically heterogeneous. In other words, if blood flow were governed only by the anatomy of the arterial network, then it becomes so heterogeneous that there exist high-flow pathways that are severely diffusion limited, and low flow pathways that are flow limited. And thus high-flow pathways are delivering venous blood that is not nearly saturated with oxygen.

Yes, our model accounts for nonlinear oxy-hemoglobin binding.

• The authors argue (in their response and in the paper) that previous studies that have suggested HPV is not significant in normoxia are not correct. The argument is not strong and is incomplete. Just because there is persistent heterogeneity of V in hyperoxia does not necessarily mean that there will be regions of persistently low PO2. It is not appropriate to include a criticism of the Arai study on this basis. The conditions that you have simulated (as well as the species) is completely different. So really your study and theirs have no relationship to each other.

We fully recognize that our perspective that HPV is active during normoxia may very well be incorrect. To our knowledge, any claim in the literature that states HPV is not active in normoxia points to this Arai study as a reference. We are not aware of any other study that independently addresses the question of the role of HPV in normoxia.

“Just because there is persistent heterogeneity of V in hyperoxia does not necessarily mean that there will be regions of persistently low PO2” – we agree, however regions with persistently low PO2 during hyperoxia is still a possibility. The entire point of our criticism of the Arai study is that we do not know the distribution or absolute values of alveolar PO2. Clinical measurements of alveolar PO2 are based on an empirical equation using blood O2, CO2, pH, and HCO3-. **Without measuring the alveolar PO2 distribution, it is difficult to quantify how hyperoxia affects alveolar PO2 distribution.**

We have moved our criticism of the Arai study from the discussion to the introduction of our manuscript. We have also clarified that the open questions that follow from the excellent Arai study serve as motivation for our study and are not a consequence of what we have learned.

Specific points:

The manuscript STILL needs a good going through for grammar and spelling

We sincerely appreciate your patience with these typos.

Abstract: You need to be clear that the model assumes that HPV is active in normoxia and that ventilation is distributed uniformly.

We agree. We have made these changes

Line 66 –you mean decreased transit time?

You are correct – thank you for catching this typo. We have corrected this in the revised manuscript.

Line 81 – I was interested in your citations to support HPV homogenizing Q and RBC transit times. I can’t see that this is claimed in ref 15 (in fact they say “progressive hypoxia leads to an increase in perfusion non-uniformity”). By ‘homogenizing’ are you just meaning that it provides a better match to V?

Thank you so much for catching this. Yes, in this study they show that at first mild hypoxia homogenizes blood flow, but progressive hypoxia leads to increasingly more heterogeneous blood flow distributions. V/Q distributions are homogenized with progressive hypoxia. We have corrected this in the revised manuscript.

For the alveolar-capillary O2 flux in Table 5, figures 6,7,8 – what period was this calculated over? This will vary throughout the transit time.

Alveolar-capillary O2 flux in Table 5, and Figs 6,7,8 is calculated by averaging quasi-steady state model simulations over 2 respiratory cycles.

I disagree with the authors’ response to my challenge that a more simple model could have been used. A simple model would indeed give the same conclusions. You could have used an arbitrary branching network with dimensions from the literature and solved a simple Poiseuille model for flow. It need not be an ODE model. By assuming a relationship between alveolar partial pressure and vessel resistance you would have predicted a decrease in flow in areas with low PO2 and an increase to other areas. It would not be very sophisticated but it would have reached the same conclusions!

Perhaps you are correct. Admittedly, we developed this model thinking about dynamic complexities of pulse wave propagation. The advantage of our ODE formulation is that it can do dynamic simulations, whereas the version you have described would only be able to represent steady state scenarios. Nevertheless, we do not believe it changes the overall conclusions of our study.

My original comment “Most pulmonary embolism does not result in decreased blood oxygen.”

My apologies: most acute pulmonary embolism (sub-massive) does not result in a major decrease in blood oxygen because of hyperventilation.

Thank you for taking the time to critically appraise our manuscript and engage in this productive review. Acute PE decreases PO2 by shunting and is partially offset by hyperventilation and tachycardia.

**Answers to Reviewer #3**

Reviewer #3: Authors have addressed the reviewer comments. Despite comments from another reviewer on the writing during previous round of reviews the manuscript still has a lot of typos and errors. It is distracting from the scientific content. For example:

Thank you for taking the time to review our manuscript, and we appreciate your patience with these typographical errors. We have corrected these that you have identified and have spent a considerable amount of time combing through to the manuscript in hopeful preparation for a smooth copyediting process.

line 23 - 'in' shouldn't be there

line 185 --- 'do' should be 'to'

equation 21 --- all subscripts are i but its not clear why the denominator has a subscript j!?

line 233 - 'oxygen oxygen'

line 319 --- 'and' upper?

line 331 -- both hypoxic and normoxic air have same % of O2 and CO2?

'airway' is randomly caps and small at multiple places in the manuscript

Resistance is accounting for viscous loss - am not sure why its labeled 'transmural resistance'!

line 469 -- goal 'of' the study

line 597-- 'that that'

check line 613 ('we not have an') and line 618 ('in a true a lung')

The Discussion after revision is now too long.

We have moved the “Physiological relevance of hypoxic pulmonary vasoconstriction” section to the introduction.
